# Delayed Subaponeurotic Fluid Collection in an Infant Without Trauma: A Literature Review and Case Report

**DOI:** 10.7759/cureus.88604

**Published:** 2025-07-23

**Authors:** Jackson L Howell, Thomas J Pittman, Christopher Coop

**Affiliations:** 1 Pediatrics, 81st Medical Group Keesler Air Force Base, Biloxi, USA; 2 Pediatrics, Keesler Air Force Base, Biloxi, USA; 3 Allergy and Immunology, Keesler Medical Center, Biloxi, USA

**Keywords:** caput succedaneum, cephalohematoma, micro-fracture, obstructed or prolonged labor, pediatrics and neonatology, subaponeurotic fluid collection - neonate, subaponeurotic (subgaleal) hemorrhage (sgh)

## Abstract

Delayed subaponeurotic fluid collections (DSFCs) are benign, focal points of fluid collection beneath the scalp’s aponeurosis. We present an infant with a complicated cesarian delivery followed by a persistent, 4-cm effusion on the patient’s posterior cranium that remained for 9 months. The exact etiology of this clinical manifestation remains uncertain, but some distinctive characteristics are important for early recognition to ensure proper interventions are taken if necessary.

## Introduction

Delayed subaponeurotic fluid collections (DSFCs) are relatively uncommon and under-reported clinical presentations in pediatric populations immediately following birth. There have been fewer than 400 documented cases worldwide to date. This number was discovered using various PubMed, Google Scholar, and pediatric journal searches. The key words used were "spontaneous subaponeurotic fluid collection (SSFC),” “delayed subaponeurotic fluid collection (DSFC),” “pediatrics and neonatology,” “obstructed or prolonged labor,” “infant cranial pathology,” and “neonatal scalp swelling.” While most children who are diagnosed with this condition typically do not exhibit any dysfunction or discomfort, the abrupt appearance within weeks or months after birth may raise alarms concerning pathology or physical abuse [1-3]. Although the etiology that causes this eruption has remained unclear, one theory is that cerebrospinal fluid (CSF) from microfractures of the skull disrupts the process of lymphatic draining, causing an accumulation of serosanguineous fluid collection [2]. While often benign, a collection left unchanged for a prolonged period without recession may lead to calcification and long-term neurological complications. This case report aimed to conduct an extensive literature review, shed light on the physical manifestation without trauma, and describe the patient's prognosis after interventions.

## Case presentation

**Figure 1:**
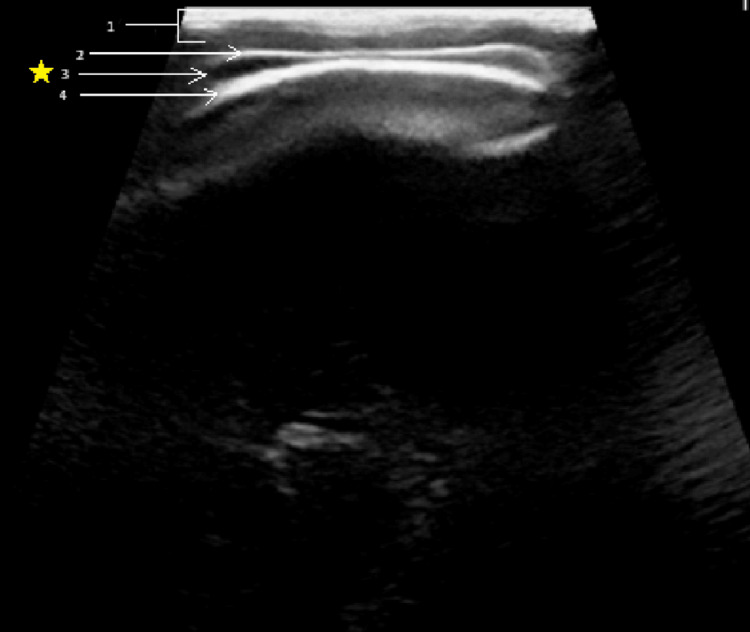
Subaponeurotic fluid collection Aponeurosis, fluid collection, and calvarium are depicted. 1) Epidermis, dermis, and subcutaneous tissue; 2) Scalp aponeurosis; 3) Subaponeurotic fluid collection; 4) Parietal bone

A female term infant, weighing 3.2 kg, was born via caesarian delivery secondary to arrest of descent. Due to the infant’s position in the birth canal, significant manipulation was required to extract the infant. After delivery, the infant was found to have a minor clavicle fracture, but otherwise had an uneventful nursery course and was discharged home with family after two days. Within 48 hours after discharge, the parents began noticing signs of occipital cranial swelling and sought out consultation at the emergency department. The infant did not show signs of pain, discomfort, or neurological dysfunction. Evaluation via ultrasound revealed a fluid collection between the periosteum and aponeurosis. Revelation of the liquid's location, as well as its ability to cross the cranial suture lines, was discovered to be consistent with a DSFC. Figure 1 shows the sonography results.

The emergency department team was highly confident in their diagnosis, alongside pediatric consultation, and did not conduct further tests. These would have included X-ray, CT scan, and hematological workup to evaluate chemical imbalance from a potential hemorrhage. The fluid collection continued to expand until approximately 12 weeks of life, growing to 4 cm, until it began to gradually resolve. After consultation with a pediatric neurosurgeon, our team was informed that although most DSFCs will have typically resolved within a few months, it is not uncommon for one to persist for a year or longer. The DSFC did eventually resolve at nine months without further complications.

## Discussion

DSFCs are unique, yet benign clinical entities that are often underreported or mistaken for other potential pathologies. Some of these include hydrocephalus, cephalohematoma, subaponeurotic hemorrhage, and caput succedaneum. These conditions may be congenital, requiring prompt neurosurgical interventions, or acquired, such as infectious meningitis or cerebral tumors [2-6]. Perilous mimickers of DSFCs are subgaleal hemorrhages, which can be potentially fatal due to their ability to grow at a rapid pace and decrease the infant’s circulating blood volume. Generally, parental or pediatric concerns warrant extensive workups, such as X-ray, blood work, and sonography, to discover the cause of the effusions. For DSFCs, sonography, combined with deduction based on fluid behavior when palpated, is the gold standard diagnostic tool for practitioners. Presentation typically occurs before the age of 12 months and resolves within 6 months [7]. The fluid on the scalp tends to be soft, absent of pain upon palpation, and unrestricted by the infant’s suture lines [5,6]. DSFCs may extend arterially, laterally, and posteriorly, as the subaponeurotic space extends from the orbits to the temporal fascia and nuchal ridge [4].

The regions affected by DSFC could be caused by trauma such as a prolonged birth without resolution, leading to a cesarian delivery, minor trauma from vacuum-assisted devices, and forceps extractions [4]. However, there have been multiple cases with minimal damage rendered upon an infant’s cranium [6,7].

Clinically, diagnosis may be made based upon neurological deficit, fluid behavior regarding the suture lines, if it is hard or soft upon palpation, its fluctuation or spread, potential calcification, and if it pits with pressure. Table 1 shows the differentiation in the clinical pathologies of the infant crown.

**Table 1:** Cranial fluid pathologies The location of the fluid collection, restrictions, palpation characteristics, and ultrasound diagnosis are the best methods of differentiating these manifestations.

Pathology	Clinical Presentation	Distinguishing Characteristics	Fluid	Resolution and Complications
Delayed Subaponeurotic Fluid Collection	May present anywhere from 1 to 18 weeks [1-4]. Fluid is located between the periosteum and aponeurosis.	Lack of neurological defects, obvious trauma during birth, and the behavior of the fluid upon palpation. It will cross the suture lines with ease and consists of soft, fluctuating fluid [4].	Cerebrospinal fluid	Varies. Will typically resolve within a week, but some cases will last longer than 1 year. Neurosurgery should be notified if over 6 months due to the risk of fluid calcification. Some literature refers to DSFC as spontaneous subaponeurotic fluid collection due to its spontaneous nature of resolving without intervention.
Caput Succedaneum	Presents shortly after delivery. A benign edema that will cross the suture lines and midline, which is typically associated with trauma after birth [8]. Extended labor, where the fetal head is subjected to pressure by the cervix or uterine walls, may cause this complication. Vacuum or forceps used for delivery have also been known causes of this manifestation.	The site of fluid accumulation and reactivity upon touch. Caput Succedaneum’s edematous presentation is located superior to the cranial suture lines, leading to a fluctuating, pitting mass that will cross the suture lines upon examination or palpation [9]. It distinguishes itself in this way from other potential, more serious etiologies such as cephalohematoma.	Serosanguinous and/or hemorrhagic	Typically resolves within 48 hours. Excellent prognosis. Rare complications: Scarring, jaundice, halo scalp, and ring alopecia.
Cephalohematoma	During an infant’s birth, shearing forces that separate the periosteum from the underlying calvarium result in a rupture of blood vessels [9]. This onset is immediately after birth and apparent upon visual and physical palpation.	Cephalohematomas occur over the parietal bones and are bound by suture lines, which restrict the fluid from crossing the midline [10-11]. Ultrasound diagnostics are best suited to separate this complication from more serious conditions such as subaponeurotic (subgaleal) hemorrhage (SGH).	Sanguineous	80% of children reabsorb the fluid collection within the first month of life [12]. Most do not have neurologic long-term deficits as the collections are not in contact with the brain parenchyma. However, anemia, infection, intracranial hemorrhage, or underlying fractures may affect 5-20% of cases [12-14].
Subaponeurotic Hemorrhage	SGH is an accumulation of blood between the epicranial aponeurosis and periosteum, caused by a rupture of the emissary veins. This is apparent at the time of birth, which is usually traumatic [15]. The blood collection may be massive, as it may extend to the anterior orbital margins, posterior nuchal ridge, and laterally temporal fascia.	This manifestation extends widely beyond the suture lines of the infant’s cranium. It is a diffuse, boggy swelling that develops on the scalp [12-15]. SGH does not have the quick reabsorption that other cranial conundrums may exhibit. Pitting edema that may shift sides will cause other physical symptoms such as ocular edema. May be diagnosed via ultrasound for quick interventions.	Hemorrhagic	SGH is the more serious condition newborns experience. Blood loss may be significant, leading to a series of problematic outcomes such as hypovolemic shock, acute anemia, coagulopathy, and death [15-16].

While most cases of DSFC resolve anywhere from 24 hours to 5 months after presentation, our patient’s fluid collection persisted for 9 months. There is an increasing risk of calcification within the scalp the longer a DSFC remains in the cranium. Neurosurgical interventions must be made to remove this, as it may cause increased risk of permanent damage as the child grows and their skull expands.

## Conclusions

DSFCs remain a clinical mystery due to their rarity and often undiagnosed nature. While DSFCs do resolve on their own, it is important to recognize their presence and improve physician awareness. Furthermore, distinguishing this clinical presentation from other more serious forms of abnormality, such as meningitis, hydrocephalus, tumors, or hemorrhage, are important to avoid unnecessary, invasive diagnostic procedures.
